# New insight into the phylogeographic pattern of *Liriodendron chinense* (Magnoliaceae) revealed by chloroplast DNA: east–west lineage split and genetic mixture within western subtropical China

**DOI:** 10.7717/peerj.6355

**Published:** 2019-02-01

**Authors:** Aihong Yang, Yongda Zhong, Shujuan Liu, Lipan Liu, Tengyun Liu, Yanqiang Li, Faxin Yu

**Affiliations:** The Key Laboratory of Horticultural Plant Genetic and Improvement of Jiangxi, Institute of Biological Resources, Jiangxi Academy of Sciences, Nanchang, Jiangxi, China

**Keywords:** Phylogeography, Subtropical China, Chloroplast DNA, *Liriodendron chinense*, Mountains, Glacial refugia

## Abstract

**Background:**

Subtropical China is a global center of biodiversity and one of the most important refugia worldwide. Mountains play an important role in conserving the genetic resources of species. *Liriodendron chinense* is a Tertiary relict tree largely endemic to subtropical China. In this study, we aimed to achieve a better understanding of the phylogeographical pattern of *L. chinense* and to explore the role of mountains in the conservation of *L. chinense* genetic resources.

**Methods:**

Three chloroplast regions (*psb*J-*pet*A, *rpl*32-*ndh*F, and *trn*K5’-*mat*K) were sequenced in 40 populations of* L. chinense* for phylogeographical analyses. Relationships among chloroplast DNA (cpDNA) haplotypes were determined using median-joining networks, and genetic structure was examined by spatial analysis of molecular variance (SAMOVA). The ancestral area of the species was reconstructed using the Bayesian binary Markov Chain Monte Carlo (BBM) method according to its geographic distribution and a maximum parsimony (MP) tree based on Bayesian methods.

**Results:**

Obvious phylogeographic structure was found in *L. chinense*. SAMOVA revealed seven groups matching the major landscape features of the *L. chinense* distribution area. The haplotype network showed three clades distributed in the eastern, southwestern, and northwestern regions. Separate northern and southern refugia were found in the Wu Mountains and Yungui Plateau, with genetic admixture in the Dalou Mountains and Wuling Mountains. BBM revealed a more ancient origin of *L. chinense* in the eastern region, with a west–east split most likely having occurred during the Mindel glacial stage.

**Discussion:**

The clear geographical distributions of haplotypes suggested multiple mountainous refugia of *L. chinense*. The east–west lineage split was most likely a process of gradual genetic isolation and allopatric lineage divergence when the Nanling corridor was frequently occupied by evergreen or coniferous forest during Late Quaternary oscillations. Hotspots of haplotype diversity in the Dalou Mountains and Wuling Mountains likely benefited from gene flow from the Wu Mountains and Yungui Plateau. Collectively, these results indicate that mountain regions should be the main units for conserving and collecting genetic resources of *L. chinense* and other similar species in subtropical China.

## Introduction

Climate oscillations during the Late Tertiary and Quaternary are believed to have had profound influences on species variation and extinction and to have shaped the population structures of relict species (Hewitt, 2000, 2004). During past climate oscillations, species retreated to refugia where conditions for survival were stable and diverse, facilitating persistence as regional biotic and abiotic environments changed (Keppel et al., 2012). Populations in such refugia functioned as initial genetic resources, and species colonized new regions until environmental conditions improved (Hewitt, 2000; Keppel et al., 2012). East Asia harbors the greatest species and phylogenetic diversity in the world due to its complex environment and topographic heterogeneity (Qian & Ricklefs, 2000). Furthermore, the strong monsoon climate and broad connection between tropical and temperate floras in southern Asia might also have helped generate the high level of phylogenetic diversity in this region (Qian, Jin & Ricklefs, 2017).

Subtropical China, located between the Qingling Mountains-Huai River line (at approx. 34 °N) and the tropical south (≤22 °N) and between 99 °E and 123 °E, is characterized by evergreen broad-leaved forests (Wu & Wu, 1998; Wang, Kent & Fang, 2007). This area consists mainly of hills and low mountains, many of which have been identified as Chinese centers of plant endemism (López-Pujol et al., 2011), and it has provided important refugia for terrestrial tree species that were not available in other regions at the same latitude. For example, the subtropical region in southern Europe and eastern North America was largely occupied by the Mediterranean Sea (Taberlet et al., 1998) and the Gulf of Mexico (Guo, Ricklefs & Cody, 1998), respectively, and mountains have played an important role in conserving the genetic resources of species and have served as refugia for paleoendemic species (Hewitt, 2000; López-Pujol et al., 2011). Indeed, the variable topography in mountainous areas provided a wide array of sheltered habitats, allowed elevational shifts of plant species to track warm interglacial/cold glacial conditions and was characterized by relatively stable environmental conditions during the Quaternary period (Hu, Hampe & Petit, 2009; Keppel et al., 2012). During glacial periods, the Qinling Mountains at the northern end of subtropical China may have acted as a barrier to cold, dry air from the north (Dong et al., 2011). Collectively, the mountains in subtropical China became some of the most important refugial locations during the Quaternary glacial period (Qiu et al., 2017).

Southeastern China was never covered by large ice sheets (Harrison et al., 2001; Ni et al., 2010), and evidence supporting the existence of multiple geographically isolated refugia for many plant species in subtropical China has accumulated (Qiu, Fu & Comes, 2011; Liu et al., 2012). Furthermore, there are no clear patterns of recolonization from the south but rather a pattern of range fragmentation (e.g., Wang et al., 2009, Wang et al., 2013, Feng et al., 2017) or only localized range expansion (e.g., Qi et al., 2012; Tian et al., 2015). Phylogeographic studies frequently suggest that the mountains in subtropical China functioned as refugia (i.e., Qiu, Fu & Comes, 2011; Liu et al., 2012; Qiu et al., 2017), dispersal corridors (e.g., Tian et al., 2015, 2018), or even genetic barriers to migration (Sun et al., 2014b; Zhang et al., 2016). As lineage differentiation and colonization mostly occurred far earlier than the last glacial maximum (LGM) (e.g., Cao et al., 2016; Zhang et al., 2016; Qiu et al., 2017), more ancient events should be considered in phylogeographic studies of subtropical organisms in China (Zhang et al., 2016). Moreover, the presence of northern refugia may have blurred a typical colonization pattern (Stewart et al., 2010), and individual biological characteristics and environmental differences may result in various phylogeographic patterns (Davis & Shaw, 2001; Petit, 2003). Hence, although the number of phylogeographic studies is rapidly increasing, given the complex topography and highly diverse flora in subtropical China, phylogeographic studies in this region remain preliminary.

The genus *Liriodendron* (Magnoliaceae) was widely distributed in the northern region of the Northern Hemisphere during the early to mid-Miocene but became extinct in Europe during the Pleistocene (Parks & Wendel, 1990), leaving the typical East Asian and eastern North American disjunct sister species *Liriodendron chinense* (Hemsl.) Sarg. and *Liriodendron tulipifera* Linn. Here, we focus on *L. chinense*, which naturally occurs in subtropical China and North Vietnam (Hao et al., 1995). In earlier research (Yang et al., 2016), we investigated the population genetics of central and peripheral populations (rear edge and leading edge) of *L. chinense* by using simple sequence repeat (SSR) markers from both the chloroplast and nuclear genomes to explore the roles of historical and present population genetics in determining the range limits of this species. However, the homology of chloroplast SSRs (cpSSRs) limited our ability to reveal the phylogeographic pattern of *L. chinense*, and a more complex phylogeographic pattern was found in the western region of *L. chinense*. Thus, in the present study, we applied three chloroplast DNA fragments (*psb*J-*pet*A, *rpl*32-*ndh*F, and *trn*K5’-*mat*K) and increased sampling to 40 populations throughout the distribution area of *L. chinense*, especially surrounding the Sichuan Basin in the western regions, aiming to draw a clearer phylogeographic pattern of *L. chinense* and to examine the role of mountains in gene conservation in subtropical China. Here, we address the following questions: (1) Is there a clear relationship between the pattern of genetic divergence and the pattern of geographic distribution? (2) Is there an obvious lineage split or obvious genetic admixture between the continuous western mountain region and scattered areas in the eastern mountain region? (3) Are there any hotspots of haplotype diversity? If such regions do exist, what is the likely underlying process creating them? The results of this study should be valuable in furthering our understanding of the present genetic distribution of *L. chinense* and in helping to formulate appropriate strategies for the conservation of *L. chinense* and other plant species with similar distribution patterns in subtropical China.

## Materials and methods

### Study species

*L. chinense* is a long-lived and monoecious tree species that inhabits subtropical forests and is the only *Liriodendron* species in Asia. *Liriodendron* trees are easily recognized by their distinctive leaves, with four lobes in most cases and a cross-cut notched or straight apex. Flowering occurs between April and May. *L. chinense* is pollinated by bees, flies, and beetles (Huang et al., 1999). Winged seeds from the aggregate fruit are dispersed by wind or gravity. The species is predominantly outcrossing, with a limited seed set and germination rate (Huang & Guo, 2002). *Liriodendron* trees prefer a temperate climate, sun or partial shade, and deep, fertile, well-drained, and slightly acidic soil.

### Population sampling

**Figure 1:**
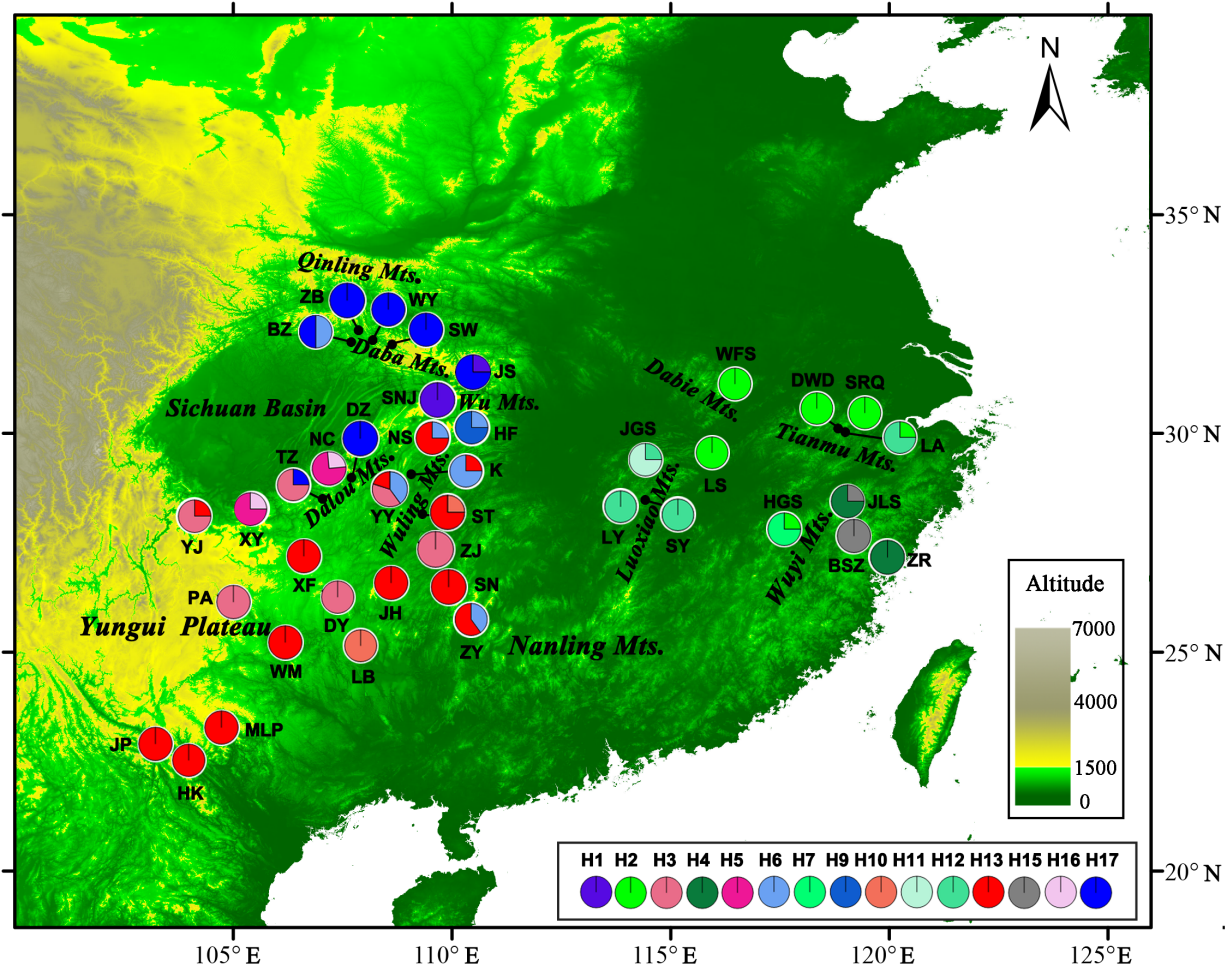
Sample locations and geographic distribution of the chloroplast (cp) DNA haplotypes detected in *Liriodendron chinense*. Pie chart size is proportional to its numbers. Detailed informations of populations and haplotypes are found in Table 1.

Considering the conservation of the chloroplast genome, we collected samples from four to five individuals from 40 populations across the entire distribution area of *L. chinense*, which included 29 populations collected in a previous study (Yang et al., 2016) and 11 populations sampled in the present study (Fig. 1; Table 1). In addition, eight individuals from four populations (Missouri, Louisiana, South Carolina, and North Carolina in the USA) of *L. tulipifera* were sampled as the outgroup. Genomic DNA was extracted from young leaves using the CTAB method (Doyle & Doyle, 1987) and stored at −20 °C.

**Table 1:** Sample localities, genetic diversity and haplotypes distributions for 40 *Liriodendron chinense* populations investigated in subtropical China.

Group/Population code	SAMOVA group	Locations	Mountain region	Elevation (m)	Longitude (° N)	Latitude (°E)	*h*	π × 10^−^^3^	Haplotypes 1(*n*)	Haplotypes 2(*n*)
**East**										
SRQ	6	Anji, Zhejiang	Tianmu Mts.	931	30.412	119.433	0.000	0.000	2(4)	2(4)
DWD	6	Jixi, Anhui	Tianmu Mts.	1,180	30.110	118.835	0.667	0.000	2(4)	2(2), 3(2)
LA	6	Lin'an, Zhejiang	Tianmu Mts.	398	30.029	118.997	1.000	0.088	2(1), 12(3)	3(1), 22(1), 23(1), 24(1)
JLS	7	Suichang, Zhejiang	Wuyi Mts.	600–952	28.361	118.858	0.500	0.083	4(3), 15(1)	9(3), 33(1)
BSZ	7	Qingyuan, Zhejiang	Wuyi Mts.	1,480	27.787	119.198	0.500	0.000	15(4)	34(3), 35(1)
ZR	7	Zherong, Fujian	Wuyi Mts.	438	27.197	119.997	0.000	0.000	4(4)	8(4)
HGS	5	Yanshan, Jiangxi	Wuyi Mts.	1,200–1,800	27.841	117.774	0.500	0.026	2(1), 7(3)	4(1), 14(3)
WFS	4	Shucheng, Anhui	Dabie Mts.	810	31.059	116.548	0.000	0.000	2(5)	4(5)
LS	4	Lushan, Jiangxi	Luoxiao Mts.	1,000–1,200	29.548	115.987	0.000	0.000	2(4)	4(4)
JGS	4	Tongshan, Hubei	Luoxiao Mts.	900–1,100	29.384	114.602	0.500	0.008	11(3), 12(1)	21(3), 26(1)
SY	5	Tonggu, Jiangxi	Luoxiao Mts.	230	28.475	114.414	0.000	0.000	12(4)	25(4)
LY	5	Liuyang, Hunan	Luoxiao Mts.	382	28.431	114.096	0.000	0.000	12(4)	26(4)
		Regional mean					0.306	0.017		
**Northwest**										
ZB	2	Zhenba, Shaanxi	Daba Mts.	904	32.351	107.863	0.000	0.000	17(5)	38(5)
WY	2	Wanyuan, Sichuan	Daba Mts.	1,122	32.126	108.183	0.000	0.000	17(3)	17(3)
BZ	2	Bazhong, Sichuan	Daba Mts.	838	32.085	107.693	0.833	0.119	6(2), 17(2)	13(2), 38(1), 39(1)
SW	2	Chengkou, Chongqing	Daba Mts.	1,404	32.030	108.628	0.000	0.000	17(4)	38(4)
SNJ	3	Shennongjia, Hubei	Wu Mts.	1,400	31.401	110.405	0.000	0.000	1(4)	1(4)
JS	3	Jianshi, Hubei	Wu Mts.	1,787	30.713	109.680	0.500	0.093	1(1), 17(3)	1(1), 37(3)
HF	3	Hefeng, Hubei	Wu Mts.	1,462	30.002	110.484	0.833	0.094	6(1), 9(3)	12(1), 16(1), 17(2)
		Regional mean					0.333	0.052		
**Southwest**										
NC	1	Nanchuan, Chongqing	Dalou Mts.	1,241	29.049	107.198	0.500	0.111	5(3), 16(1)	10(3), 36(1)
DZ	1	Daozhen, Guizhou	Dalou Mts.	1,537	28.983	107.698	0.500	0.000	17(4)	38(3), 39(1)
TZ	1	Tongzi, Guizhou	Dalou Mts.	1,579	28.500	107.038	0.833	0.107	3(3), 17(1)	12(1), 16(1), 17(2)
XY	1	Xuyong, Sichuan	Yungui Plateau	1,278	28.197	105.492	0.000	0.000	5(4)	10(4)
YJ	1	Yanjin, Yunnan	Yungui Plateau	783	28.067	104.135	0.833	0.097	3(3), 13(1)	6(2), 7(1), 31(1)
XF	1	Xifeng, Guizhou	Yungui Plateau	1,470	27.119	106.623	0.000	0.000	13(4)	32(4)
JH	1	Jianhe, Guizhou	Yungui Plateau	1,000–1,300	26.497	108.690	0.000	0.000	13(4)	29(4)
DY	1	Duyun, Guizhou	Yungui Plateau	1,368	26.270	107.364	0.000	0.000	3(4)	7(4)
PA	1	Pu’an, Guizhou	Yungui Plateau	1,614	26.095	105.023	0.000	0.000	3(4)	7(4)
WM	1	Wangmo, Guizhou	Yungui Plateau	1,295	25.407	106.133	0.000	0.000	13(4)	32(4)
LB	1	Libo, Guizhou	Yungui Plateau	849	25.226	107.868	0.000	0.000	19(4)	10(4)
MLP	1	Malipo, Yunnan	Yungui Plateau	1,683	23.137	104.754	0.000	0.000	13(4)	30(4)
JP	1	Jinping, Yunnan	Yungui Plateau	1,595	22.812	103.257	0.000	0.000	13(5)	30(5)
HK	1	Hekou, Yunnan	Yungui Plateau	454	22.585	103.913	0.000	0.000	13(4)	30(4)
NS	1	Enshi, Hubei	Wuling Mts.	1,539	29.682	109.716	0.500	0.107	6(1), 13(3)	11(1), 32(3)
K	1	Longshan, Hunan	Wuling Mts.	1,200	29.067	109.067	0.700	0.128	6(3), 13(1)	11(3), 28(1)
YY	1	Youyang, Chongqing	Wuling Mts.	1,329	28.968	108.656	0.800	0.089	3(2), 6(2), 13(1)	7(2), 11(2), 28(1)
ST	1	Songtao, Guizhou	Wuling Mts.	882	28.157	109.319	0.833	0.000	10(1), 13(3)	18(1), 28(1), 29(2)
ZJ	1	Zhijiang, Hunan	Wuling Mts.	341	27.597	109.638	0.000	0.000	13(4)	29(4)
SN	1	Suining, Hunan	Xuefeng Mts.	505	26.448	110.108	0.000	0.000	3(4)	5(4)
ZY	1	Ziyuan, Guangxi	Nanling Mts.	1,181	25.850	110.363	0.600	0.128	6(3), 13(2)	11(3), 29(2)
		Regional mean					0.280	0.033		
West		Regional mean					0.295	0.038		
Whole range		Species mean					0.298	0.032		

**Notes:**

*h*, total genetic diversity; π, total nucleotide diversity; Haplotypes 1, haplotypes excluding SSR variations; Haplotypes 2, haplotypes including SSR variations.

### Genotyping and sequencing

We designed eight noncoding plastid DNA region primers as suggested by Shaw et al. (2007) based on the *L. chinense* chloroplast genome; amplification was performed using DNA from eight individuals from eight distant populations of *L. chinense.* Of the primers, *psb*J*-pet*A and *rpl*32*-ndh*F were found to be the most variable and stable. In addition, we also used three cpDNA primers (*psb*A*-trn*H, *trn*L intron*-trn*F, and *trn*K5’*-mat*K) reported by Fetter (2014) for *L. tulipifera*. Only *trn*K5’*-mat*K amplification produced polymorphic and stable results; in contrast, the *psb*A*-trn*H fragment contained too many SSRs, reducing the accuracy of the sequencing results. Additionally, *trn*L intron*-trn*F was too conservative. Hence, *psb*J*-pet*A, *rpl*32*-ndh*F, and *trn*K5’*-mat*K were selected for a large-scale survey of haplotype variation in *Liriodendron* (Table 2). The cpDNA regions were amplified under the protocols reported by Fetter (2014). All sequences were generated using an ABI3730xl DNA sequencer (Applied Biosystems, Foster City, CA, USA), aligned using DNASTAR Lasergene V.7.1 and then proofed and corrected manually. All sequences were deposited in GenBank under accession numbers MH717109–MH717129 (*psb*J-*pet*A), MH717130–MH717142 (*rpl*32-*ndh*F), and MH717143–MH717150 (*trn*K5’-*mat*K) (Table 2). The haplotype for each individual was identified by combining three cpDNA fragments.

**Table 2:** Molecular marker primers used in this study and their sources.

Primers Pairs (5′–3′)	Primer name	Variations	Genebank no.	Reference
F: ATGATTCTAGGAGGGATTACR: CTTTTACGGTTCATATTCTGGATT	*psb*J*-pet*A	21	MH717109–MH717129	Newly designed
F: ATCTTCATATCTTCATTACGAR: ATTGTTTCCGATTCACCAG	*rpl*32-*ndh*F	13	MH717130–MH717142	Newly designed
F: CGGGTTGCTAACTCAACGGR: GTTCGTAAAAAATCGATCCA	*trn*K5’-*mat*K	8	MH717143–MH717150	Fetter (2014)

### Population genetic and phylogeographical analysis

The haplotype diversity (*h*; Nei, 1987) and nucleotide diversity (*Pi*; Tajima, 1983) were estimated for each population using DnaSP version 5.0 (Librado & Rozas, 2009). The species total genetic diversity (*h*_T_ and *v*_T_) and population genetic diversity (*h*_S_ and *v*_S_) were calculated by PermutCpSSR 2.0 (Pons & Petit, 1996). Genetic diversity between the scattered eastern mountain regions (E) and continuous western mountain habitats (W), including regions with genetic and habitat heterogeneity (northwest, NW; southwest, SW), were compared using a *t*-test or Mann–Whitney *U* test, depending on the distribution of each parameter, in the SPSS 13.0 program (SPSS Inc., Chicago, IL, USA). Nonparametric correlations (Spearman) between genetic diversity (*h* and π) in the western regions and latitude were also tested.

To quantify the variation in cpDNA sequences among populations and genetic clusters in the eastern and western regions, we performed hierarchical analysis of molecular variance (AMOVA) with 10,000 permutations in Arlequin version 3.1 (Excoffier, Laval & Schneider, 2005). Spatial analysis of molecular variance (SAMOVA) was applied to identify clusters of genetically similar populations. Geographically homogeneous populations were clustered into a user-defined number of groups (*K*) with a simulated annealing approach to maximize the proportion of total genetic variance observed between groups (*F*_CT_). SAMOVA was conducted with SAMOVA 1.0 (Dupanloup, Schneider & Excoffier, 2002) by using 100 simulated annealing processes for each value of *K* from *K* = 2 to *K* = 20.

The presence of phylogeographic structure was determined by whether the value of *R*_ST_ (considering the mutational distances between haplotypes) was significantly higher than that of *G*_ST_ (depending on the frequencies of haplotypes), with 1,000 random permutations in PermutCpSSR version 2.0 (Pons & Petit, 1996). Intraspecific genealogies of haplotypes were constructed using a median-joining network in Network 5.0 (Bandelt, Forster & Röhl, 1999), with all indels and single- or multiple-base substitutions treated as one-step mutations and weighted equally. As the SSR regions were too variable, SSRs were excluded from the network and the later maximum parsimony (MP) construction, though we included SSR regions in other analyses.

Two selective neutrality tests, namely, Tajima’s *D* (Tajima, 1989) and Fu’s *F*_S_ (Fu, 1997), were performed to infer potential population growth and expansion in Arlequin v3.1 (Excoffier, Laval & Schneider, 2005), and significantly negative values indicated population expansion (Fu, 1997). Pairwise mismatch distributions were analyzed to infer the historical demography of *L. chinense* and each (sub)clade. We calculated the expected frequency based on a population growth-decline model. The sum of squared deviations (SSDs) between the observed and expected mismatch distributions and the raggedness index (*H*_Rag_; Harpending, 1994) were calculated with 1,000 parametric bootstrap replicates to test the null hypothesis of spatial expansion using Arlequin ver. 3.1. Furthermore, Bayesian Skyline Plots (BSPs) were used to test the hypothesis of demographic expansion by assessing the time variation in effective population size. The BSPs were constructed in BEAST ver. 2.1.3 (Bouckaert et al., 2014), and settings were referred to Amarilla et al. (2015). The substitutions rate was set as 1.0–3.0 × 10^−9^ per site per year (s/s/y). Two independent runs were carried out for a chain length of 1 × 10^8^ with a burn-in of 10%. The results of each run were visualized using Tracer v 1.7 (Rambaut et al., 2018) to ensure that stationarity and convergence had been reached (ESS > 200). All the demographic expansion tests were also performed for each of the three genetically heterogeneous regions.

### Divergence between cpDNA lineages and ancestral area reconstructions

Phylogenetic relationships among the cpDNA haplotypes were reconstructed using the MP method with *L. tulipifera* as the outgroup. We conducted MP analyses in Mega 6 (Tamura et al., 2007) and used heuristic searches with the random addition of sequences (1,000 replicates) and tree bisection–reconnection branch swapping selected; gaps were treated as complete deletions. We calculated bootstrap values using 1,000 replicates of the original dataset, and five random additional sequences were added to each replicate.

A molecular clock model was applied to estimate the cpDNA lineage divergence time. The rate constancy of cpDNA haplotype evolution in *L. chinense* was first evaluated by Tajima’s relative rate tests (Tajima, 1993) in MEGA 6 (Tamura et al., 2007) using *L. tulipifera* as the outgroup. Then, the time since divergence (*T*) of the haplotype lineages was inferred from their net pairwise sequence divergence per base pair (*d*_A_) using the Kimura 2-parameter model. *T* was calculated as *T* = *d*_A_/2μ, where μ is the rate of nucleotide substitutions (Nei & Kumar, 2000); μ is derived from the pairwise genetic distance and an assumed *T* between *L. chinense* and *L. tulipifera* of 14.15 Mya (Nie et al., 2008).

To reconstruct the geographical ancestral area of *L. chinense*, a Bayesian binary Markov chain Monte Carlo (MCMC) (BBM) analysis was performed in RASP v3.0 (Yu et al., 2015; http://mnh.scu.edu.cn/soft/blog/RASP) using trees retained from Mega 6 (see above). *L. tulipifera* was used as an outgroup for our ancestral state reconstructions. Three geographic regions were defined according to the distribution pattern of *L. chinense* (Hao et al., 1995) and adjusted based on the presumed refugia, i.e., EA, East; SW, southwest; and NW, Northwest. The number of maximum areas at each node was set to four. One thousand trees produced by Mega 6 were used for BBM analyses. We set the root distribution to null, applied 10 MCMC chains with the JC + G model run for 106 generations, and sampled the posterior distribution every 100 generations.

## Results

### Haplotype distribution and genetic diversity

For the cpDNA dataset, the *psb*J*-pet*A, *rpl*32*-ndh*F, and *trn*K5’*-mat*K sequences, consisting of a consensus length of 2,080 bp with 23 nucleotide substitutions, were combined and aligned. In addition, one multiple-base substitution (12 bp in length), three indels (8–9 bp in length), and four SSRs were also identified (Supplementary data in Table S1). A total of 43 haplotypes were identified from these 32 polymorphisms (h1–h43, Table S1). Thirty-seven haplotypes were found in *L. chinense,* and six haplotypes were identified in *L. tulipifera*. No haplotype was shared by the two sister species. The geographical distribution of the haplotypes and their frequencies within populations are illustrated in Table 1.

CpDNA haplotypes were distributed relatively evenly over the entire range of *L. chinense*. No haplotype was obviously dominant, and 22 of the 37 haplotypes were unique to a single population (Table 1). In the western region, h38 was the most frequent haplotype in the northwest, and h30 was found only in the southwestern range. Each population in the Yungui Plateau contained only one haplotype, except for YJ, which was located in the northwestern Yungui Plateau and harbored three haplotypes. Population HF in the Wu Mountains and YY in the Wuling Mountains also harbored three haplotypes. In the eastern region, only h4 occurred in more than two populations. Most populations contained one to two haplotypes, except for LA, located in the Tianmu Mountains, which contained four haplotypes, largely due to the high degree of variation in the SSR regions (Table 1). At the species level, three cpDNA regions exhibited high haplotype diversity (v_T_ = 0.972, *h*_T_ = 0.970) and nucleotide diversity (π_T_ = 1.756 × 10^−3^), though the average population haplotype diversity was relatively low (*h*_S_ = 0.300, *v*_S_ = 0.195). The unbiased haplotype diversity (*h*) for each population was zero to one (LA), and the nucleotide diversity ranged from 0 to 0.128 × 10^−3^ (ZY and K). No significant deficiency in genetic diversity was detected in the scattered eastern mountain regions (*P* > 0.05, Table 1). In the continuous western mountain regions, genetic diversity was not significantly correlated with latitude (*h*: Spearman *r* = 0.340, *P* = 0.076; π: Spearman *r* = 0.318, *P* = 0.099), but hotspots of haplotype diversity occurred at mid-latitudes close to the current northern edge, around the Wu Mountains, Dalou Mountains, and Wuling Mountains. The number of haplotypes was clearly low in the southwestern range (Fig. S1).

#### Population genetic structure of L. chinense

**Figure 2:**
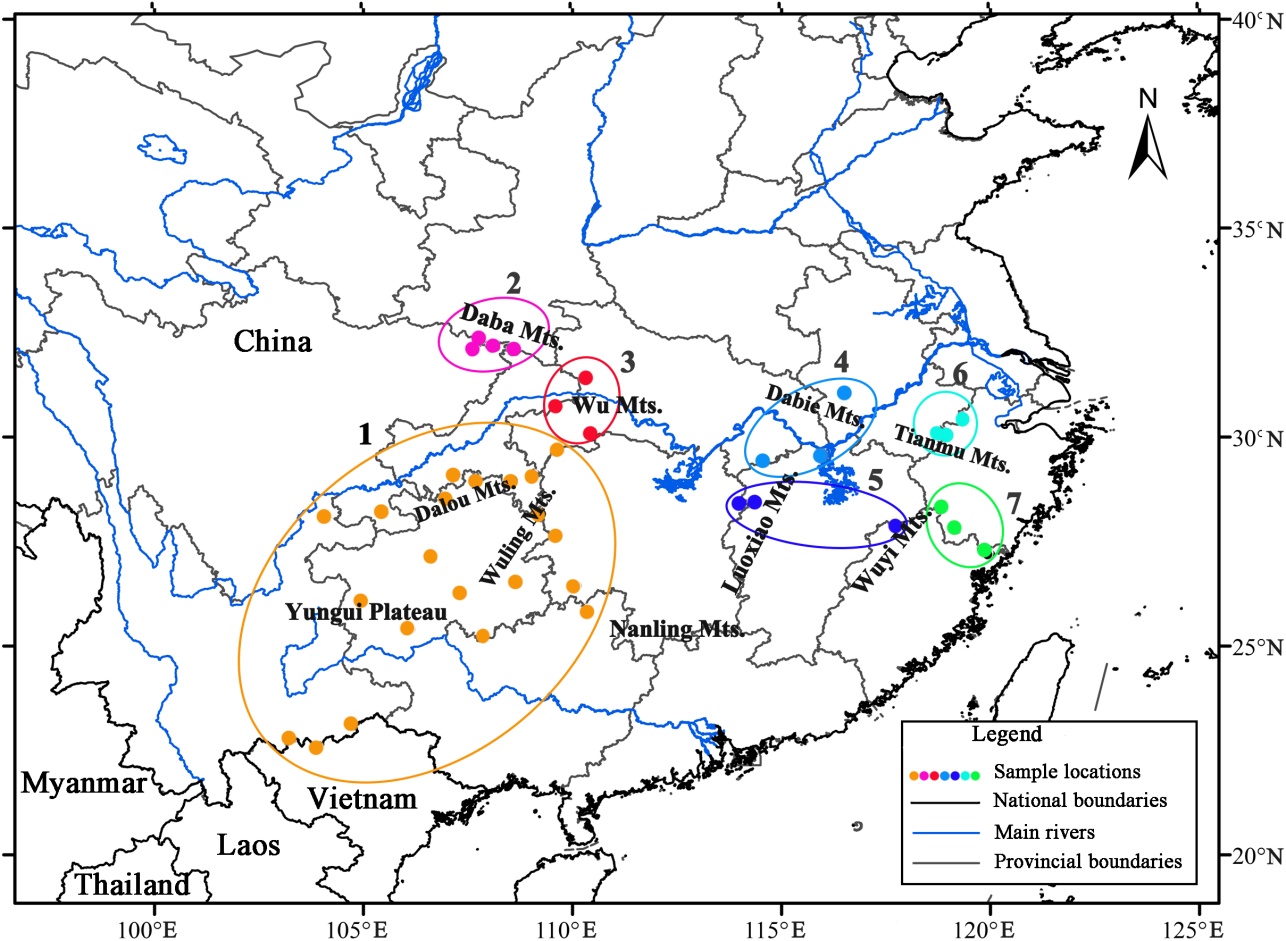
Results of spatial analysis of molecular variance analysis (SAMOVA, *K* = 7) on *Liriodendron chinense* populations in subtropical China.

AMOVA revealed strong population genetic structure at the species level (*F*_ST_ = 0.747, *P* < 0.001). Hierarchical AMOVA revealed that 17.74% of the variation between the two groups and 56.97% of the total variation in cpDNA of the species was distributed within the eastern and western regions (Table 3). By exploring the behavior of the indices *F*_CT_ (minimized) and *F*_SC_ (maximized) for different values of *K*, the best population grouping scheme for a set of sample populations can be identified in SAMOVA (Dupanloup, Schneider & Excoffier, 2002). In the present study, SAMOVA revealed a discontinuous increase in *F*_CT_ and a discontinuous decrease in *F*_SC_ when *K* increased from 2 to 20 (Fig. S2). However, *F*_CT_ reached its first peak and *F*_SC_ was relatively low when *K* = 7, and the trends in *F*_CT_ and *F*_SC_ reversed when *K* = 8. Although *K* = 11 was still acceptable, two groups were subdivided; thus, we used *K* = 7 as the best grouping scheme. The composition of groups corresponded well with the geographical distribution (Fig. 2). The first group had a geographically wide distribution and was composed of all the populations in the southwest. Specifically, the unique ‘island’ in the western range (Hao et al., 1995) of the Nanling Mountains was genetically consistent with populations in the continuous western region (the “belt”). Populations from the Daba Mountains composed group 2, and the three populations located in the Wu Mountains formed group 3. The populations in groups 2 and 3 exclusively formed the northwestern clade. Populations in the eastern region were obviously divided into four groups (4–7), with group 6 containing populations from the Tianmu Mountains in the northeastern region and group 7 including populations in the eastern Wuyi Mountains in the southeastern region. The populations located in the northern and central regions (Luoxiao Mountains) were divided into two groups (Fig. 2).

**Table 3:** Results of AMOVA test of the population genetic structure in *Liriodendron chinense*.

Source of variation	d.f.	Percentage of variation	*P*	*F*_ST_
Among groups	1	17.74	< 0.001	
Among populations within groups	38	56.97	< 0.001	
Within populations	124	25.29	< 0.05	0.747*

**Note:**

* *P* < 0.001, 1,000 permutations.

#### Phylogeographic pattern of *L. chinense*

**Figure 3:**
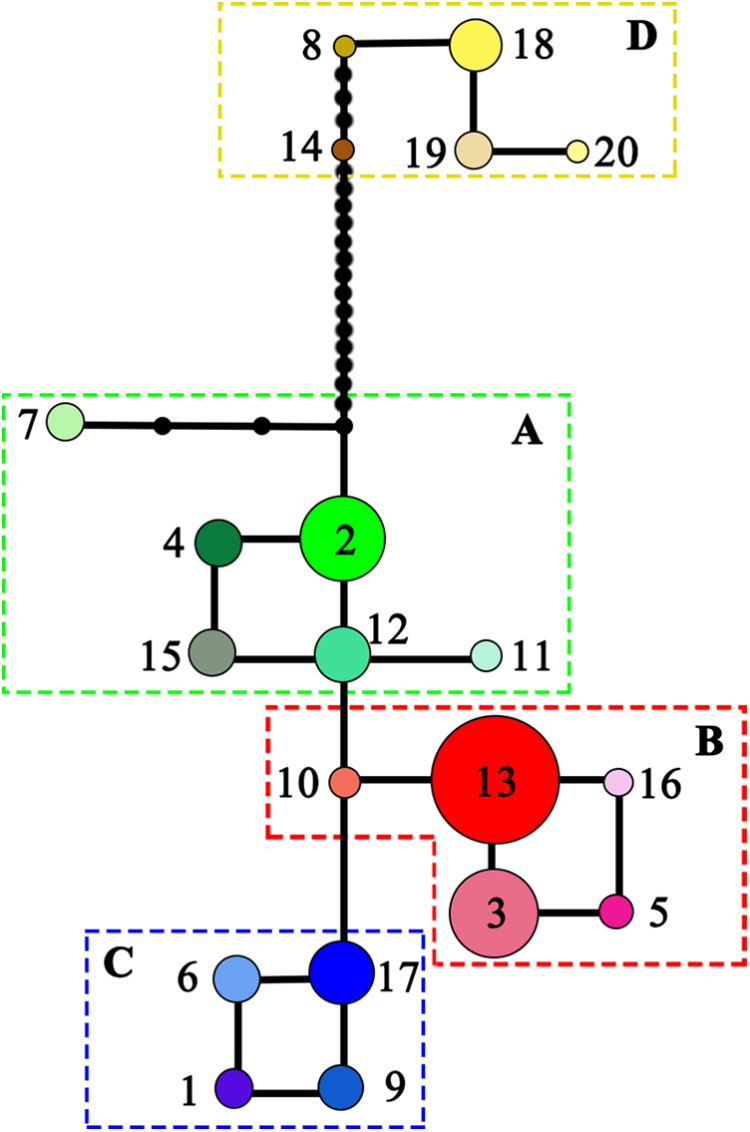
Median-joining network of the 20 haplotypes detected in *Liriodendron* *chinense* (clades A–C) and *Liriodendron tulipifera* (clade D). Black dots indicated the number of mutational steps.

*N*_ST_ was significantly higher than *G*_ST_ (*N*_ST_ = 0.799, *G*_ST_ = 0.691; *P* < 0.001), indicating significant phylogeographic structure across the range of *L. chinense*. Four main clades (A–D) were revealed by the three cpDNA regions in the median-joining network (Fig. 3). Haplotypes from *L. tulipifera* formed clade D. Clade A was composed of all the haplotypes from the eastern and central populations in the eastern region. Haplotypes in clades B and C were specific to the western region. All populations in the southwestern region harbored haplotypes from clade B, whereas all populations from the northwestern region contained haplotypes in clade C; populations in the midwestern region usually contained haplotypes from both the B and C clades (Fig. 1; Table 1). Notably, clade A connected with clade D and showed a close relationship with *L. tulipifera*, and clade C appeared as a tip clade. Among these haplotypes, H2 in clade A was the fewest mutational steps from *L. tulipifera*, and this haplotype was widely distributed in the northern part of the eastern region, including the Tianmu Mountains, Dabie Mountains, and Lu Mountains.

**Figure 4:**
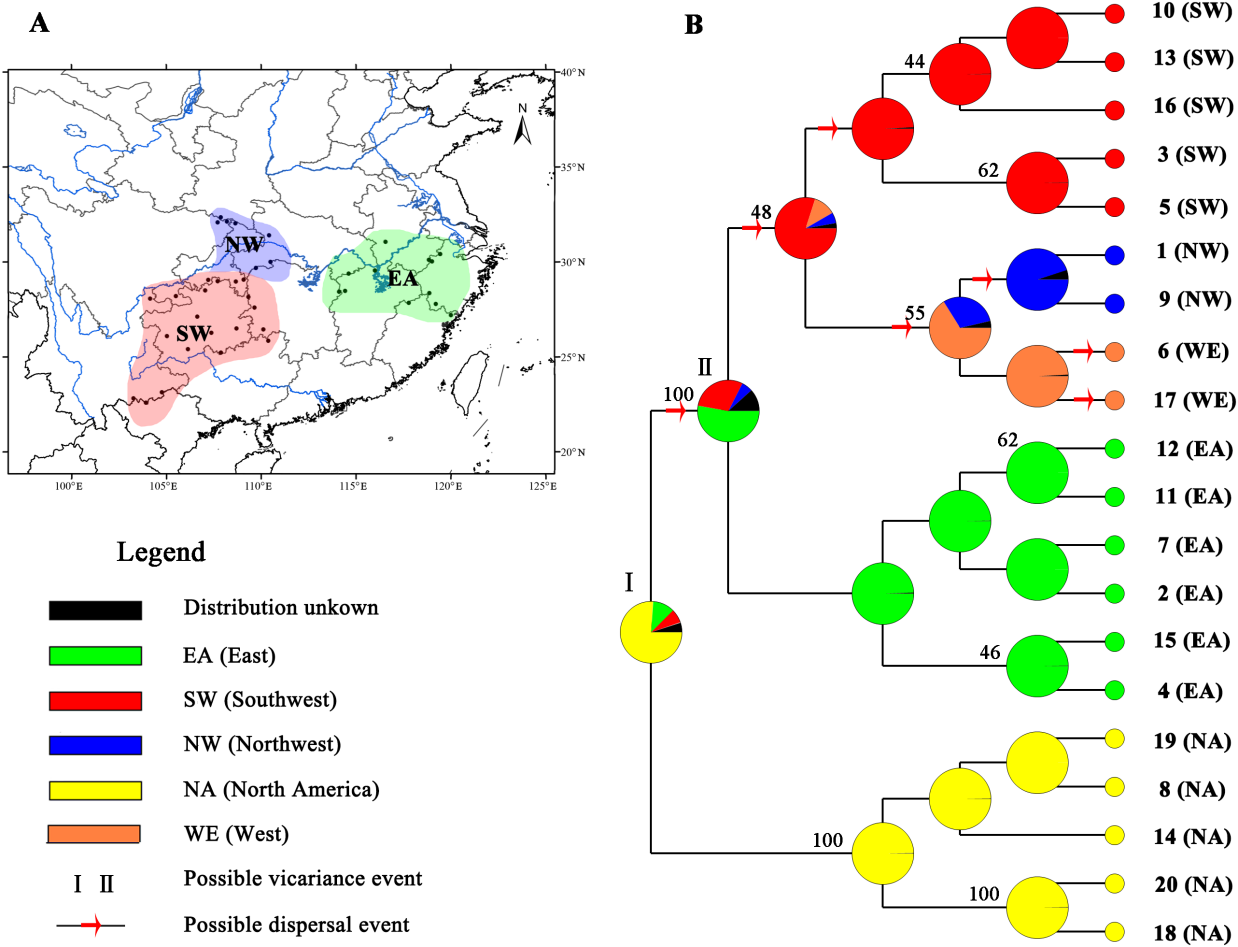
Ancestral area reconstructions based on the Bayesian binary Markov chain Monte Carlo (BBM) method implemented in RASP using the MP chronogram of *Liriodendron chinense*. (A) The insert map shows major floristic divisions in Subtropical China. (B) Maximum parsimony (MP) tree of the 20 haplotypes detected in Liriodendron. Pie charts of each node illustrate the marginal probabilities for each alternative ancestral area derived from BBM with the maximum area number set to four. The number above the branches indicate bootstraps support value above 40. The colour key identifies possible ancestral ranges at different nodes. Possible dispersal events are indicated by red arrows.

The MP tree showed a pattern similar to that in the haplotype network (Fig. 4). Obvious eastern and western clades were found, and the western clade was further divided into two subclades: the southwestern and northwestern subclades. Relative rate constancy tests suggested equal rates among the cpDNA haplotypes of *L. chinense* and *L. tulipifera* (all *P* > 0.05), indicating that the clock-like evolution model (Tajima, 1993) was applicable for *L. chinense*. Based on a divergence time between the *Liriodendron* sister species of 14.15 Ma, a divergence time between the eastern and western regions of *L. chinense* of 0.433 Ma (95% CI = 0.0254–0.841 Ma) was suggested.

### Demographic analysis and ancestral area reconstructions

None of the estimates of Tajima’s *D* were significant for any cpDNA (sub)clade of *L. chinense*. Although all Fu’s *F*_S_ values were positive, only the Fu’s *F*_S_ over the entire range (*P* < 0.01) was significant. The observed mismatch distributions of haplotypes for each (sub)clade failed to reject the spatial expansion model in most cases (SSD, *H*_Rag_ values, *P* > 0.05; Table 4), except for the SW lineage, with a significant SSD and *H*_Rag_ statistic (*P* < 0.05). The results of BSPs showing the estimated changes in effective population size over time for the whole and each (sub)clade were concordant with the results from the neutrality tests. They showed that *L. chinense* had a constant population size during the last 15,000 years (Figs. S3A–D).

**Table 4:** Results of mismatch distribution analysis and neutrality tests for pooled populations of lineages and subclades of *Liriodendron chinense*.

Lineage/subclade	*SSD*	*P*	*H*_Rag_	*p*	Fu’s *Fs*	*P*	Tajima’s *D*	*P*
East lineage	0.00836	0.693	0.0144	0.824	−1.021	0.396	−0.0185	0.543
West lineage	0.0128	0.088	0.0350	0.108	−3.337	0.168	1.596	0.915
Southwest subclade	0.0225	0.027	0.0750	0.005	−2.175	0.255	1.473	0.926
Northwest subclade	0.0201	0.388	0.0739	0.477	−1.438	0.213	1.354	0.913
Whole range	0.00280	0.460	0.0110	0.548	−10.296	0.009	0.140	0.599

Based on the topology of the interspecific chronogram, the BBM analysis of ancestral distribution areas (Fig. 4) supported a likely ancient vicariance event (node I, NA, and EA) of two sister species. A subsequent vicariance event (node II) between populations in eastern (EA) and southwestern (SW) China was found. This event was then followed by dispersal from the east (EA) to the southwest (SW). No obvious divergence event was detected between NW and SW; instead, several dispersal events were evident. Recent colonization events from the southwest (SW) to the northwest (NW) and the reverse (NW to SW) were inferred, based on the genetic admixture (H6 and H17) in the adjacent mountains (Figs. 1 and 4), with multiple expansions at different times.

## Discussion

### East–west lineage split and the role of the Nanling Mountains

By investigating cpDNA sequence variation, an obvious west–east lineage split across subtropical China was found for *L. chinense*, and this *L. chinense* west–east genetic divergence was further shown by the phenotypic divergence between the eastern and western regions, such as divergence in petal color and lobed leaf number (Hao et al., 1995). Furthermore, according to the results of the molecular phylogenetic analysis based on RAD-seq, the eastern and western populations of *L. chinense* formed two clades (Zhong et al., 2019). These results, coupled with those from some other species (Shi et al., 2014; Sun et al., 2014a; Wang et al., 2015; Feng et al., 2017), emphasize the role of allopatric genetic divergence in shaping the phylogeographic patterns of plant species in subtropical China, providing new insight into the dual role of the Nanling Mountains in facilitating a west–east lineage split of temperate plant species in subtropical China.

Since the late Tertiary climate oscillation, repeated allopatry has caused frequent speciation (Hewitt, 2004). The breaking off of Beringia during the late Tertiary global cooling event induced species splits between Asia and North America (Milne & Abbott, 2002); *L. chinense* and *L. tulipifera* were among the species that diverged (Parks & Wendel, 1990). To the best of our knowledge, phylogeographic studies in subtropical China have reinforced the allopatric speciation hypothesis for species diversity, and the phylogeographic divergence in subtropical China is mostly attributed to the tectonic and climate changes during the Quaternary (Qiu et al., 2017). Specifically, the uplift of the Himalayan–Tibetan Plateau and the subsequent formation of Asian monsoons generated a split of the southwestern clade (southwestern Yungui Plateau) from the clades in other regions for many plant species, such as *Tetrastigma hemsleyanum* (Southwest China *vs.* Central and East China: 5.07 Ma; Wang et al., 2015), *Taxus wallichiana* (Sino-Himalayan *vs*. others: 6.58 Ma, Tonkin Bay region *vs.* Sino-Japanese forest subkingdom: 3.84 Ma; Gao et al., 2007), and *Euptelea pleiosperma* (West China *vs.* Central/East China: 1.66 Ma; Cao et al., 2016). Nonetheless, although *L. chinense* was also spread across different geological formations and two climate types in the Yungui Plateau, no lineage differences were found (Figs. 1 and 2). In contrast, the observed west–east divergence in *L. chinense* occurred in the eastern Yungui Plateau and Sino-Japanese forest subkingdom region (Wu & Wu, 1998), which have relatively consistent geological and climatic conditions. Compared with the long-term isolation and ancient allopatric divergence of *Quercus spinosa* (Southwest China *vs*. Central and East China: 25.67 Ma, Feng et al., 2017) in subtropical China, the probable divergence time of *L. chinense* between the east and west was estimated to have been approximately 0.433 Ma, which closely coincides with the early Mindel glaciation (approx. 0.30–0.45 Ma). Although recently occurring and not as pronounced, a west–east lineage split has also been detected in many other species, such as *Fagus engleriana* (Lei et al., 2012), *Castanopsis eyrei* (Shi et al., 2014), and *Castanopsis fargesii* (Sun et al., 2014a). We argue that the west–east lineage split was likely a process driven by gradual geographic isolation and allopatric genetic divergence. However, the process that underlies the divergence that occurs along a geographic feature, such as between the western (eastern Yungui Plateau) and eastern regions, remains unclear, though it is likely the result of the dual corridor and barrier roles played by the Nanling Mountains.

The Nanling Mountains, stretching from the west to the east for more than 1,000 km in subtropical China, have been recognized as an important corridor for the eastward migration of East Asiatic flora, referred to as the Nanling corridor (Wang, 1992). Recent phylogeographic studies have shown that the Nanling Mountains acted as a stepping stone and dispersal corridor for species, facilitating the exchange of genetic resources between the eastern Yungui Plateau and the eastern mountain regions in subtropical China during the Late Quaternary (e.g., Gao et al., 2007; Wang et al., 2009; Tian et al., 2015, 2018). In fact, according to the particular location of the Nanling Mountains and the westward dispersal event from the eastern regions inferred from the cpDNA-based BBM analysis, we assumed that the Nanling Mountains may have also contributed to the ancient dispersal of *L. chinense* to the eastern Yungui Plateau.

However, the Nanling corridor was frequently inhabited by coniferous or evergreen forest during the Quaternary glacial and interglacial periods (Harrison et al., 2001; Qiu, Fu & Comes, 2011). In the case of *L. chinense*, although there is a wide confidence range, the divergence time of the west–east lineage split (0.0254–0.841 Ma) occurred before the LGM (~0.018 Ma), and the Nanling Mountains were climatically unsuitable during the LGM (Yang et al., 2016). An inescapable consequence of the interruption of gene flow, the west–east lineage split may have developed due to gradual geographic isolation. In view of biological differences in the response to climate change among species, the Nanling Mountains may have functioned as a “filter” (such as the East China Sea land bridge during the Quaternary, Qiu et al., 2017) for different species. Hence, the west–east lineage split only partially occurred in some species, such as *F. engleriana* (Lei et al., 2012), *C. eyrei* (Shi et al., 2014), and *C. fargesii* (Sun et al., 2014a), but the split is not currently observed in species that are widely distributed in the Nanling Mountains and are able to produce sufficient gene flow between the eastern mountains and the Yungui Plateau (i.e., *Taxus wallichiana*, Gao et al., 2007; *Sargentodoxa cuneata*, Tian et al., 2015; and *Tetrastigma hemsleyanum*, Wang et al., 2015).

### “Melting pot” due to admixture in the continuous western mountain region

Coexistence of the northern refugium in the eastern Daba/Wu Mountain regions and the southern refugium in the Yungui Plateau has also been found in many other subtropical species (i.e., *Taxus wallichiana*, Gao et al., 2007; *Ligularia hodgsonii*, Wang et al., 2013; *Tetrastigma hemsleyanum*, Wang et al., 2015; *Euptelea*, Cao et al., 2016; and *Platycarya*, Wan et al., 2017). Although not stressed by authors, genetic admixture of southern and northern refugia is frequently found, and the Wuling Mountains, which are located in the contact region, usually were the center of genetic admixture of many plant species, such as *Taxus wallichiana* (Gao et al., 2007), *Tetrastigma hemsleyanum* (Wang et al., 2015), and *Emmenopterys henryi* (Zhang et al., 2016).

For *L. chinense*, the Wuling Mountains and the Dalou Mountains are the present distribution center (Hao et al., 1995) and genetic diversity center (Fig. 1; Fig. S1), respectively. The high genetic diversity of this species may be explained by a center of ancient origin or genetic admixture from the northern and southern refugia. Coalescent theory predicts that older alleles will occupy the interior nodes of a haplotype network (Posada & Crandall, 2001). Nevertheless, none of the *L. chinense* populations there harbored ancient haplotypes from both clades. More precisely, the populations in the Dalou Mountains (NC, DZ, and TZ) contained the ancestral haplotype H17 from clade C (northwestern clade) and tip haplotypes (H3, H5, and H16) from clade B (southwestern clade). In contrast, populations in the Wuling Mountains (YY, K, and NS) all exclusively contained ancestral haplotype H13 from clade B and one tip haplotype, H6, from clade C (Figs. 1 and 3). Therefore, there were no areas where diversity was created. In fact, species usually shifted northward or southward to track warm interglacial or cold glacial conditions during the Quaternary climate oscillation (Hewitt, 2000). Increased diversity was more likely to occur in zones of admixture produced by redistribution of the genetic diversity created in refugia (Petit, 2003). For example, in *E. henryi,* genetic admixture of the northern lineage with the southern lineage is more likely explained by both introgressions following secondary contact and incomplete lineage sorting due to recent postglacial divergence during glacial periods (Zhang et al., 2016). Collectively, these findings suggest that the genetic diversity centers of *L. chinense* in the Wuling Mountains and Dalou Mountains were not the primary locations of ancient refugia but more likely a “melting pot” due to the admixture of divergent lineages from northern or southern refugia.

The next question, then, is what the underlying process is. In contrast to wave-like migration fronts, expansion from local refugia followed by subsequent population admixture is more likely to be characteristic of postglacial forest development (Hu, Hampe & Petit, 2009). In western subtropical China, the Dalou Mountains and the Wuling Mountains are located in the northern and northeastern portions of the Yungui Plateau, respectively, and they are geographically connected with the Yungui Plateau. We argue that these mountains can receive haplotypes from the eastern Yungui Plateau refugium by occasional capture because the expansion of *L. chinense* in the Yungui Plateau refugium appears to have been imperceptible. In view of the facts that clade C did not show a "star-like" pattern of population expansion and that the mismatch test and neutral test rejected an expansion pattern in the Yungui Plateau (Table 4), gene flow from the Yungui Plateau to adjacent mountains was weak and repeated several times, showing no obvious signals of sudden expansion. For example, despite the high cpDNA diversity in *Populus adenopoda* (Fan et al., 2018), the Yungui Plateau may have contributed little to its recolonization of northern and eastern areas. The most likely scenario for *L. chinense* was that the southern refugia resources had a pan-distribution pattern in the Yungui Plateau and the species reached its northern range limit in the Dalou Mountains (NC, DZ, and TZ) and Wuling Mountains (YY, K, and NS), where it encountered northwestern genetic resources (TZ, DZ, YY, K, and NS).

Glacial refugial populations at mid-to-high latitudes sometimes appear to play a greater role in the postglacial development of forest biomes than those inferred from fossil pollen records (Hu, Hampe & Petit, 2009). Similarly, in *L. chinense*, the southward introgression of northern refugial resources greatly contributed to the genetic admixture in the Dalou Mountains and Wuling Mountains. The northern refugium was centered in the Wu Mountains, where all the haplotypes in clade C were located (Table 1; Fig. 1). In contrast, in the Dalou Mountains (DZ and TZ), the haplotypes were almost exclusively H17, and the Wuling Mountains (YY, K, and NS) harbored only tip haplotypes from clade C. Although not significant, demographic analysis partially suggested weak expansion in the northwestern subclade (Table 4). Therefore, the Wu Mountains may have contributed to the southward colonization of *L. chinense* to the adjacent Dalou Mountains and Wuling Mountains (Figs. 1 and 2). This pattern was also found in other species, for example, in *Tetrastigma hemsleyanum* (Wang et al., 2015). Haplotypes in the Dalou Mountains (CC4 and CC5) and northern Wuling Mountains (CC6 and CC8) were also derived from the Wu Mountains (CC7, haplotype 9). Furthermore, genetic resources from the Wu Mountain region may have also contributed to genetic admixture in the Nanling Mountains (ZY, Fig. 1) or even farther east in the Luoxiao Mountains (*Tetrastigma hemsleyanum*, Wang et al., 2015). Given these results, we argue that genetic diversity in the Dalou Mountains and Wuling Mountains is largely due to the frequent contact of southern and northern refugium resources in response to past glacial and interglacial cycles.

### Mountain refugia and implications for conservation

Evidence supporting the presence of multiple geographically isolated refugia of plant species in subtropical China without an obvious range shift to the south during the LGM has accumulated (reviewed by Qiu, Fu & Comes, 2011 and Liu et al., 2012), such as that found in *E. cavaleriei* (Wang et al., 2009), *F. engleriana* (Lei et al., 2012), *Platycarya* (Wan et al., 2017) and *P. adenopoda* (Fan et al., 2018). In the case of *L. chinense*, a “refugia within refugia” scenario (c.f. Gómez & Lunt, 2007) of multiple localized glacial refugia across the range has been suggested (Yang et al., 2016). Similarly, in the present study, the results of three cpDNA sequence variations indicated the presence of five mountain-based refugia across the range of *L. chinense*. These refugia were located in the Wu Mountains (northwest), Yungui Plateau (southwest), Luoxiao Mountains (center), Tianmu Mountains (northeast) and Wuyi Mountains (southeast), falling almost entirely within the main mountain ranges (Figs. 1 and 2).

These mountain regions reportedly act as important refugia for many plant species. For instance, the Wu Mountains were a large endemism center (López-Pujol et al., 2011) and functioned as an important refugium for relict tree species (e.g., *E. cavaleriei*, Wang et al., 2009; *Tetracentron sinense*, Sun et al., 2014b; and *E. henryi*, Zhang et al., 2016). The Tianmu Mountains formed a northern refugium counterpart to the Wu Mountains, especially for many relict species (e.g., *Ginkgo biloba*, Gong et al., 2008; and *F. engleriana*, Lei et al., 2012). The Wuyi Mountains were previously reported to serve as an important refugium (e.g., Wang et al., 2009; Sun et al., 2014a; Wan et al., 2017). We also found divergent haplotypes in the Wuyi Mountains regions, and special attention should be paid to the haplotype divergence of *L. chinense* between the eastern and western ranges of the Wuyi Mountains (Figs. 1 and 3), which has also been found in *E. cavaleriei* (Wang et al., 2009) and *E. henryi* (Zhang et al., 2016).

From the perspective of conservation biology, regions possessing different haplotypes should be targeted to maintain the maximum amount of genetic diversity. Hence, the main mountain refugia located in the scattered eastern and continuous western mountain regions should be included. Furthermore, in view of their high degree of harbored genetic diversity, the Daba and Wuling Mountains need special protection. Although *L. chinense* is listed as an endangered species in China (Fu & Jin, 1992), fortunately, most populations are currently protected in nature reserves or areas used as fengshui forests by local villagers (Tang et al., 2013). Due to the high genetic heterogeneity of *L. chinense*, *in situ* conservation priorities should target populations from the five main mountain refugial regions. For *ex situ* conservation, reintroduction, seed banking, and collection of germplasm resources should be implemented to reserve the unique germplasm resources of *L. chinense*.

## Conclusion

A west–east lineage split across subtropical China was found for *L. chinense*. This pattern is different from the clear lineage divergence in the southwestern (southwestern Yugui Plateau)–central China split, which was mainly caused by the ancient uplift of the Himalayan–Tibetan Plateau and the formation of Asian monsoons (e.g., Wang et al., 2015; Cao et al., 2016; Zhang et al., 2016). The west–east split examined in the present study is more likely a process of gradual genetic isolation and allopatric lineage divergence, especially in some environmentally sensitive plant species, when the Nanling corridor was frequently inhabited by coniferous or evergreen forest during the Quaternary climate oscillation (Harrison et al., 2001; Qiu, Fu & Comes, 2011).

In the continuous western mountain region, genetic resources from the southern refugium and northern refugium combined in the Dalou Mountains and the Wuling Mountains. The contact regions became the regions of high genetic diversity and modern distribution centers of *L. chinense.* In the scattered eastern mountain regions, genetic differentiation likely occurred as the habitat gradually fragmented in each mountain region. This large-scale phylogeographical investigation demonstrates that *L. chinense* was structured into several distinct groups. Each group showed a marked mountain-associated pattern, falling almost entirely within the main mountain ranges, many of which were hypothesized to be areas of former glacial refugia (reviewed by Liu et al., 2012 and Qiu et al., 2017) unique to the Wu Mountains, Yungui Plateau, Tianmu Mountains, Luoxiao Mountains, and Wuyi Mountains.

Collectively, these results indicate that mountain regions should be the main genetic resource conservation and collection units for *L. chinense*. Our results for *L. chinense* contribute to a growing body of evidence regarding the role of mountains in forming refuges for subtropical species and the different phylogeographic patterns in separate mountain regions. To obtain a better understanding of the phylogeographic pattern in subtropical China, we should emphasize studies on species with different biological characteristics in subtropical China.

## Supplemental Information

10.7717/peerj.6355/supp-1 Supplemental Information 1 Description of haplotypes in *Liriodendron* from three chloroplast DNA fragments combined. All sequences are compared to the reference haplotype H1.

10.7717/peerj.6355/supp-2 Supplemental Information 2 Bubble diagram of population haplotype diversity (*h*) of *Liriodendron chinense* in western mountain region. The size of each bubble was consistent with relative value of haplotype diversity, and the hollow circles represent the value of 0.

10.7717/peerj.6355/supp-3 Supplemental Information 3 Results of spatial analysis of molecular variance analysis (SAMOVA, *K* = 2–20) on *Liriodendron chinense* populations in subtropical China. *K* refers to the number of predefined groups used in the analyses.

10.7717/peerj.6355/supp-4 Supplemental Information 4 Bayesian skyline plot showing the estimated effective population size by region. The *x*-axis measures time in millions of years and the *y*-axis is the scaled effective population size. The thick solid line is the mean estimate, and the gray areas show the 95% HPD limits. (a) Whole range; (b) East lineage; (c) Southwest subclade; (d) Northwest subclade.

10.7717/peerj.6355/supp-5 Supplemental Information 5 CpDNA sequences (*psb*J*-pet*A, *rpl*32*-ndh*F, and *trn*K5’*-mat*K ) in *Liriodendron*.

## Additional Information and Declarations

### DNA Deposition

The following information was supplied regarding the deposition of DNA sequences: The sequences are accessible via GenBank accession numbers MH717109–MH717129 (psbJ-petA), MH717130–MH717142 (rpl32-ndhF), and MH717143–MH717150 (trnK5’-matK).

### Data Availability

The following information was supplied regarding data availability: The raw data are available in Table 1 and Table S1. The locations of all the populaitons used are listed in Table 1, along with haplotypes and their numbers for each population. The three cpDNA fragments used in this study are available in GenBank. All the haplotypes with sequence differences are available in Table S1.

## References

[ref-1] Amarilla LD, Anton AM, Chiapella JO, Manifesto MM, Angulo DF, Sosa V (2015). *Munroa argentina*, a grass of the South American transition zone, survived the Andean uplift, aridification and glaciations of the Quaternary. PLOS ONE.

[ref-2] Bandelt HJ, Forster P, Röhl A (1999). Median-joining networks for inferring intraspecific phylogenies. Molecular Biology and Evolution.

[ref-3] Bouckaert R, Heled J, Kuhnert D, Vaughan T, Wu CH, Xie D, Suchard MA, Rambaut A, Drummond AJ (2014). BEAST 2: a software platform for Bayesian evolutionary analysis. PLOS Computational Biology.

[ref-4] Cao Y-N, Comes HP, Sakaguchi S, Chen L-Y, Qiu Y-X (2016). Evolution of East Asia's Arcto-Tertiary relict *Euptelea* (Eupteleaceae) shaped by Late Neogene vicariance and Quaternary climate change. BMC Evolutionary Biology.

[ref-5] Davis MB, Shaw RG (2001). Range shifts and adaptive responses to Quaternary climate change. Science.

[ref-6] Dong Y, Zhang G, Neubauer F, Liu X, Genser J, Hauzenberger C (2011). Tectonic evolution of the Qinling orogen, China: Review and synthesis. Journal of Asian Earth Sciences.

[ref-7] Doyle JJ, Doyle JL (1987). A rapid DNA isolation procedure for small quantities of fresh leaf tissue. Phytochemical Bulletin.

[ref-8] Dupanloup I, Schneider S, Excoffier L (2002). A simulated annealingapproach to define the genetic structure of population. Molecular Ecology.

[ref-9] Excoffier L, Laval G, Schneider S (2005). Arlequin ver. 3.0: an integrated software package for population genetics data analysis. Evolutionary Bioinformatics.

[ref-10] Fan L, Zheng H, Milne RI, Zhang L, Mao K (2018). Strong population bottleneck and repeated demographic expansions of *Populus adenopoda* (Salicaceae) in subtropical China. Annals of Botany.

[ref-11] Feng L, Zhang YP, Chen XD, Yang J, Zhou T, Bai GQ, Yang J, Li ZH, Peng CI, Zhao GF (2017). Allopatric divergence, local adaptation, and multiple Quaternary refugia in a long-lived tree (*Quercus spinosa*) from subtropical China. BioRxiv.

[ref-12] Fetter KC (2014). Migration, adaptation, and speciation—A post-glacial history of the population structure, phylogeography, and biodiversity of *Liriodendron tulipifera* L. (Magnoliaceae).

[ref-13] Fu YX (1997). Statistical tests of neutrality of mutations against population growth, hitchhiking and background selection. Genetics.

[ref-14] Fu LK, Jin JM (1992). China plant red data book—Rare and endangered plants.

[ref-15] Gao LM, Mőller M, Zhang X-M, Hollingsworth ML, Liu J, Mill RR, Gibby M, Li D-Z (2007). High variation and strong phylogeographic pattern among cpDNA haplotypes in *Taxus wallichiana* (Taxaceae) in China and North Vietnam. Molecular Ecology.

[ref-16] Gómez A, Lunt DH, Weiss S, Ferrand N (2007). Refugia within refugia: catterns of phylogeographic concordance in the Iberian Peninsula. Phylogeography of Southern European Refugia.

[ref-17] Gong W, Chen C, Dobeš C, Fu C-X, Koch MA (2008). Phylogeography of a living fossil: pleistocene glaciations forced *Ginkgo biloba* L. (Ginkgoaceae) into two refuge areas in China with limited subsequent postglacial expansion. Molecular Phylogenetics and Evolution.

[ref-18] Guo Q, Ricklefs RE, Cody ML (1998). Vascular plant diversity in eastern Asia and North America: historical and ecological explanations. Botanical Journal of the Linnean Society.

[ref-19] Hao RM, He SA, Tang SJ, Wu SP (1995). Geographical distribution of *Liriodendron chinense* in China and its significance. Journal of Plant Resources and Environment.

[ref-20] Harpending HC (1994). Signature of ancient population growth in a low-resolution mitochondrial DNA mismatch distribution. Human Biology.

[ref-21] Harrison SP, Yu G, Takahara H, Prentice IC (2001). Palaeovegetation (Communications arising): diversity of temperate plants in east Asia. Nature.

[ref-22] Hewitt G (2000). The genetic legacy of the Quaternary ice ages. Nature.

[ref-23] Hewitt GM (2004). Genetic consequences of climatic oscillations in the Quaternary. Philosophical Transactions of the Royal Society B: Biological Sciences.

[ref-24] Hu FS, Hampe A, Petit RJ (2009). Paleoecology meets genetics: deciphering past vegetational dynamics. Frontiers in Ecology and the Environment.

[ref-25] Huang SQ, Guo YH (2002). Variation of pollination and resource limitation in a low seed-set tree, *Liriodendron chinense* (Magnoliaceae). Botanical Journal of the Linnean Society.

[ref-26] Huang SQ, Guo YH, Pan MQ, Chen JK (1999). Floral syndrome and insect pollination of *Liriodendron Chinense* (in Chinese with English abstract). Acta Botanica Sinica.

[ref-27] Keppel G, Van Niel KP, Wardell-Johnson GW, Yates CJ, Byrne M, Mucina L, Schut AGT, Hopper SD, Franklin SE (2012). Refugia: identifying and understanding safe havens for biodiversity under climate change. Global Ecology and Biogeography.

[ref-28] Lei M, Wang Q, Wu Z-J, López-Pujol J, Li D-Z, Zhang Z-Y (2012). Molecular phylogeography of Fagus engleriana (Fagaceae) in subtropical China: limited admixture among multiple refugia. Tree Genetics & Genomes.

[ref-29] Librado P, Rozas J (2009). DnaSP v5: a software for comprehensive analysis of DNA polymorphism data. Bioinformatics.

[ref-30] Liu J-Q, Sun Y-S, Ge X-J, Gao L-M, Qiu Y-X (2012). Phylogeographic studies of plants in China: advances in the past and directions in the future. Journal of Systematics and Evolution.

[ref-31] López-Pujol J, Zhang F-M, Sun H-Q, Ying T-S, Ge S (2011). Centres of plant endemism in China: places for survival or for speciation?. Journal of Biogeography.

[ref-32] Milne RI, Abbott RJ (2002). The origin and evolution of tertiary relict floras. Advances in Botanical Research.

[ref-33] Nei M (1987). Molecular evolutionary genetics.

[ref-34] Nei M, Kumar S (2000). Molecular evolution and phylogenetics.

[ref-35] Ni J, Yu G, Harrison SP, Prentice IC (2010). Palaeovegetation in China during the late Quaternary: Biome reconstructions based on a global scheme of plant functional types. Palaeogeography, Palaeoclimatology, Palaeoecology.

[ref-36] Nie Z-L, Wen J, Azuma H, Qiu Y-L, Sun H, Meng Y, Sun W-B, Zimmer EA (2008). Phylogenetic and biogeographic complexity of Magnoliaceae in the Northern Hemisphere inferred from three nuclear data sets. Molecular Phylogenetics and Evolution.

[ref-37] Parks CR, Wendel JF (1990). Molecular divergence between Asian and North American species of *Liriodendron* (Magnoliaceae) with implications forinterpretation of fossil floras. American Journal of Botany.

[ref-39] Petit RJ (2003). Glacial refugia: Hotspots but not melting pots of genetic diversity. Science.

[ref-40] Pons O, Petit RJ (1996). Measuring and testing genetic differentiation with ordered versus unordered alleles. Genetics.

[ref-41] Posada D, Crandall KA (2001). Intraspecific gene genealogies: tree grafting into networks. Trends in Ecology and Evolution.

[ref-42] Qi XS, Chen C, Comes HP, Sakaguchi S, Liu YH, Tanaka N, Sakio H, Qiu YX (2012). Molecular data and ecological niche modelling reveal a highly dynamic evolutionary history of the East Asian Tertiary relict *Cercidiphyllum* (Cercidiphyllaceae). New Phytologist.

[ref-43] Qian H, Jin Y, Ricklefs RE (2017). Phylogenetic diversity anomaly in angiosperms between eastern Asia and eastern North America. Proceedings of the National Academy of Sciences of the United States of America.

[ref-44] Qian H, Ricklefs RE (2000). Large-scale processes and the Asian bias in species diversity of temperate plants. Nature.

[ref-45] Qiu YX, Fu CX, Comes HP (2011). Plant molecular phylogeography in China and adjacent regions: Tracing the genetic imprints of Quaternary climate and environmental change in the world’s most diverse temperate flora. Molecular Phylogenetics and Evolution.

[ref-46] Qiu Y, Lu Q, Zhang Y, Cao Y (2017). Phylogeography of East Asia’s Tertiary relict plants: current progress and future prospects. Biodiversity Science.

[ref-47] Rambaut A, Drummond AJ, Xie D, Baele G, Suchard MA (2018). Posterior summarisation in Bayesian phylogenetics using Tracer 1.7. Systematic Biology.

[ref-48] Shaw J, Lickey EB, Schilling EE, Small RL (2007). Comparison of whole chloroplast genome sequences to choose noncoding regions for phylogenetic studies in angiosperms: the tortoise and the hare III. American Journal of Botany.

[ref-49] Shi MM, Michalski SG, Welk E, Chen XY, Durka W, Carine M (2014). Phylogeography of a widespread Asian subtropical tree: genetic east-west differentiation and climate envelope modelling suggest multiple glacial refugia. Journal of Biogeography.

[ref-50] Stewart JR, Lister AM, Barnes I, Dalen L (2010). Refugia revisited: individualistic responses of species in space and time. Proceedings of the Royal Society B: Biological Sciences.

[ref-51] Sun Y, Hu H, Huang H, Vargas-Mendoza CF (2014a). Chloroplast diversity and population differentiation of *Castanopsis fargesii* (Fagaceae): a dominant tree species in evergreen broad-leaved forest of subtropical China. Tree Genetics and Genomes.

[ref-52] Sun Y, Moore MJ, Yue L, Feng T, Chu H, Chen S, Ji Y, Wang H, Li J, Carine M (2014b). Chloroplast phylogeography of the East Asian Arcto-Tertiary relict *Tetracentron sinense* (Trochodendraceae). Journal of Biogeography.

[ref-53] Taberlet P, Fumagalli L, Wust-Sauc AG, Cosson JF (1998). Comparative phylogeography and postglacial colonization routes in Europe. Molecular Ecology.

[ref-54] Tajima F (1983). Evolutionary relationship of DNA sequences in finite populations. Genetics.

[ref-55] Tajima F (1989). Statistical method for testing the neutral mutation hypothesis by DNA polymorphism. Genetics.

[ref-56] Tajima F (1993). Simple methods for testing the molecular evolutionary clock hypothesis. Genetics.

[ref-57] Tamura K, Dudley J, Nei M, Kumar S (2007). MEGA4: Molecular Evolutionary Genetics Analysis (MEGA) software version 4.0. Molecular Biology and Evolution.

[ref-70] Tang CQ, Yang Y, Ohsawa M, Momohara A, Mu J, Robertson K (2013). Survival of a tertiary relict species, Liriodendron chinense (Magnoliaceae), in southern China, with special reference to village fengshui forests. American Journal of Botany.

[ref-58] Tian S, Kou Y, Zhang Z, Yuan L, Li D, López-Pujol J, Fan D, Zhang Z (2018). Phylogeography of *Eomecon chionantha* in subtropical China: the dual roles of the Nanling Mountains as a glacial refugium and a dispersal corridor. BMC Evolutionary Biology.

[ref-59] Tian S, Lei S-Q, Hu W, Deng L-L, Li B, Meng Q-L, Soltis DE, Soltis PS, Fan D-M, Zhang Z-Y (2015). Repeated range expansions and inter-/postglacial recolonization routes of *Sargentodoxa cuneata* (Oliv.) Rehd. et Wils. (Lardizabalaceae) in subtropical China revealed by chloroplast phylogeography. Molecular Phylogenetics and Evolution.

[ref-60] Wan Q, Zheng Z, Huang K, Guichoux E, Petit RJ (2017). Genetic divergence within the monotypic tree genus *Platycarya* (Juglandaceae) and its implications for species’ past dynamics in subtropical China. Tree Genetics & Genomes.

[ref-61] Wang WT (1992). On some distribution patterns and some migration routes found in the eastern Asiatic region. Acta Phytotaxonomica Sinica.

[ref-62] Wang J, Gao P, Kang M, Lowe AJ, Huang H (2009). Refugia within refugia: the case study of a canopy tree (*Eurycorymbus cavaleriei*) in subtropical China. Journal of Biogeography.

[ref-63] Wang J-F, Gong X, Chiang Y-C, Kuroda C, Linder P (2013). Phylogenetic patterns and disjunct distribution in *Ligularia hodgson*ii Hook. (Asteraceae). Journal of Biogeography.

[ref-64] Wang Y-H, Jiang W-M, Comes HP, Hu FS, Qiu Y-X, Fu C-X (2015). Molecular phylogeography and ecological niche modelling of a widespread herbaceous climber, *Tetrastigma hemsleyanum* (Vitaceae): insights into Plio-Pleistocene range dynamics of evergreen forest in subtropical China. New Phytologist.

[ref-65] Wang X-H, Kent M, Fang X-F (2007). Evergreen broad-leaved forest in Eastern China: its ecology and conservation and the importance of resprouting in forest restoration. Forest Ecology and Management.

[ref-66] Wu Z, Wu S, Zhang AL, Wu SG (1998). A proposal for a new floristic kingdom (realm): the E. Asiatic Kingdom, its delineation and characteristics. Floristic Characteristics and Diversity of East Asian Plants: Proceedings of the First International Symposium of Floristic Characteristics and Diversity of East Asian Plants.

[ref-67] Yang A, Dick CW, Yao X, Huang H (2016). Impacts of biogeographic history and marginal population genetics on species range limits: a case study of *Liriodendron chinense*. Scientific Reports.

[ref-68] Yu Y, Harris AJ, Blair C, He X (2015). RASP (Reconstruct Ancestral State in Phylogenies): a tool for historical biogeography. Molecular Phylogenetics and Evolution.

[ref-69] Zhang Y-H, Wang IJ, Comes HP, Peng H, Qiu Y-X (2016). Contributions of historical and contemporary geographic and environmental factors to phylogeographic structure in a Tertiary relict species, *Emmenopterys henryi* (Rubiaceae). Scientific Reports.

[ref-71] Zhong Y, Yang A, Liu S, Liu L, Li Y, Wu Z, Yu F (2019). RAD-seq data point to a distinct split in *Liriodendron* (Magnoliaceae) and obvious east-west genetic divergence in *L. chinense*. Forests.

