# Supplementary material for: New insight into the phylogeographic pattern of Liriodendron chinense (Magnoliaceae) revealed by chloroplast DNA: east–west lineage split and genetic mixture within western subtropical China
Source: PeerJ. 2019 Feb 1;7:e6355. doi: 10.7717/peerj.6355 (PMC6361005; doi:10.7717/peerj.6355)
Supplement: Supplemental Information 1 — All sequences are compared to the reference haplotype H1. [file peerj-07-6355-s001.docx]

Table S1 Description of haplotypes in *Liriodendron* from three chloroplast DNA fragments combined.

| Haplotype | | *psb*J-*pet*A | | | | | |  | *rpl*32-*ndh*F | | | | | | | | | | | | | |  | *trn*K5’-*mat*K | | | | | | | |  | A | |  | B |  | C |
| --- | --- | --- | --- | --- | --- | --- | --- | --- | --- | --- | --- | --- | --- | --- | --- | --- | --- | --- | --- | --- | --- | --- | --- | --- | --- | --- | --- | --- | --- | --- | --- | --- | --- | --- | --- | --- | --- | --- |
| Nucleotide position (bp) | | 51 | 116 | 348 | 366 | 431 | 460 |  | 33 | 34 | 63 | 107 | 178 | 179 | 214 | 343 | 368 | 560 | 621 | 663 | 683 | 726 |  | 91 | 136 | 164 | 171 | 240 | 405 | 477 | 564 |  | 4  5  3 | 5  5  7 |  | 6  3  4 |  | 3  8  9 |
| H1 | h1 | * | φ | C | A | G | G |  | A | G | G | § | T | A | G | C | T | A | G | G | T | C |  | G | A | G | T | C | 4 | C | T |  | 10 | 14 |  | 11 |  | 9 |
| H2 | h2 | · | · | · | · | · | · |  | C | A | · | · | · | · | · | · | · | · | · | · | · | · |  | · | · | A | · | · | · | · | · |  | 10 | 14 |  | 11 |  | 9 |
|  | h3 | · | · | · | · | · | · |  | C | A | · | · | · | · | · | · | · | · | · | · | · | · |  | · | · | A | · | · | · | · | · |  | 10 | 13 |  | 11 |  | 9 |
|  | h4 | · | · | · | · | · | · |  | C | A | · | · | · | · | · | · | · | · | · | · | · | · |  | · | · | A | · | · | · | · | · |  | 9 | 11 |  | 11 |  | 9 |
| H3 | h5 | · | · | · | · | · | · |  | C | A | · | · | · | · | · | · | · | · | · | · | · | · |  | · | · | · | · | · | - | · | · |  | 10 | 17 |  | 11 |  | 9 |
|  | h6 | · | · | · | · | · | · |  | C | A | · | · | · | · | · | · | · | · | · | · | · | · |  | · | · | · | · | · | - | · | · |  | 10 | 15 |  | 14 |  | 9 |
|  | h7 | · | · | · | · | · | · |  | C | A | · | · | · | · | · | · | · | · | · | · | · | · |  | · | · | · | · | · | - | · | · |  | 10 | 15 |  | 11 |  | 9 |
| H4 | h8 | · | · | · | · | · | · |  | C | A | · | · | · | · | · | · | · | · | · | T | · | · |  | · | · | A | · | · | · | · | · |  | 10 | 18 |  | 11 |  | 9 |
|  | h9 | · | · | · | · | · | · |  | C | A | · | · | · | · | · | · | · | · | · | T | · | · |  | · | · | A | · | · | · | · | · |  | 10 | 17 |  | 10 |  | 9 |
| H5 | h10 | · | · | · | · | · | · |  | C | A | · | - | · | · | · | · | · | · | · | · | · | · |  | · | · | · | · | · | - | · | · |  | 10 | 15 |  | 11 |  | 9 |
| H6 | h11 | · | · | · | · | · | · |  | C | · | · | · | · | · | · | · | · | · | · | · | · | · |  | · | · | · | · | · | · | · | · |  | 10 | 14 |  | 11 |  | 9 |
|  | h12 | · | · | · | · | · | · |  | C | · | · | · | · | · | · | · | · | · | · | · | · | · |  | · | · | · | · | · | · | · | · |  | 10 | 13 |  | 11 |  | 9 |
|  | h13 | · | · | · | · | · | · |  | C | · | · | · | · | · | · | · | · | · | · | · | · | · |  | · | · | · | · | · | · | · | · |  | 10 | 12 |  | 11 |  | 9 |
| H7 | h14 | · | - | A | · | · | · |  | C | A | A | · | G | · | · | · | · | · | · | · | · | · |  | · | · | A | · | · | · | · | · |  | 10 | 12 |  | 12 |  | 9 |
| H8 | h15 | · | - | · | G | T | A |  | C | A | · | · | · | C | A | T | G | · | T | · | G | A |  | A | · | A | C | A | · | T | G |  | 15 | 12 |  | 11 |  | 11 |
| H9 | h16 | # | · | · | · | · | · |  | · | · | · | · | · | · | · | · | · | · | · | · | · | · |  | · | · | · | · | · | · | · | · |  | 10 | 15 |  | 11 |  | 9 |
|  | h17 | # | · | · | · | · | · |  | · | · | · | · | · | · | · | · | · | · | · | · | · | · |  | · | · | · | · | · | · | · | · |  | 10 | 14 |  | 11 |  | 9 |
| H10 | h18 | # | · | · | · | · | · |  | C | A | · | · | · | · | · | · | · | · | · | · | · | · |  | · | · | · | · | · | · | · | · |  | 10 | 18 |  | 11 |  | 9 |
|  | h19 | # | · | · | · | · | · |  | C | A | · | · | · | · | · | · | · | · | · | · | · | · |  | · | · | · | · | · | · | · | · |  | 10 | 16 |  | 10 |  | 9 |
| H11 | h21 | # | · | · | · | · | · |  | C | A | · | · | · | · | · | · | · | · | · | · | · | · |  | · | G | A | · | · | · | · | · |  | 9 | 11 |  | 11 |  | 9 |
| H12 | h22 | # | · | · | · | · | · |  | C | A | · | · | · | · | · | · | · | · | · | · | · | · |  | · | · | A | · | · | · | · | · |  | 10 | 15 |  | 11 |  | 9 |
|  | h23 | # | · | · | · | · | · |  | C | A | · | · | · | · | · | · | · | · | · | · | · | · |  | · | · | A | · | · | · | · | · |  | 10 | 14 |  | 11 |  | 9 |
|  | h24 | # | · | · | · | · | · |  | C | A | · | · | · | · | · | · | · | · | · | · | · | · |  | · | · | A | · | · | · | · | · |  | 10 | 12 |  | 11 |  | 9 |
|  | h25 | # | · | · | · | · | · |  | C | A | · | · | · | · | · | · | · | · | · | · | · | · |  | · | · | A | · | · | · | · | · |  | 9 | 12 |  | 10 |  | 9 |
|  | h26 | # | · | · | · | · | · |  | C | A | · | · | · | · | · | · | · | · | · | · | · | · |  | · | · | A | · | · | · | · | · |  | 9 | 11 |  | 11 |  | 9 |
| H13 | h28 | # | · | · | · | · | · |  | C | A | · | · | · | · | · | · | · | · | · | · | · | · |  | · | · | · | · | · | - | · | · |  | 10 | 18 |  | 11 |  | 9 |
|  | h29 | # | · | · | · | · | · |  | C | A | · | · | · | · | · | · | · | · | · | · | · | · |  | · | · | · | · | · | - | · | · |  | 10 | 17 |  | 11 |  | 9 |
|  | h30 | # | · | · | · | · | · |  | C | A | · | · | · | · | · | · | · | · | · | · | · | · |  | · | · | · | · | · | - | · | · |  | 10 | 16 |  | 11 |  | 8 |
|  | h31 | # | · | · | · | · | · |  | C | A | · | · | · | · | · | · | · | · | · | · | · | · |  | · | · | · | · | · | - | · | · |  | 10 | 15 |  | 14 |  | 9 |
|  | h32 | # | · | · | · | · | · |  | C | A | · | · | · | · | · | · | · | · | · | · | · | · |  | · | · | · | · | · | - | · | · |  | 10 | 15 |  | 12 |  | 9 |
| H14 | h27 | # | · | · | G | T | A |  | C | A | · | · | · | C | A | T | G | · | T | · | G | A |  | A | · | G | C | A | - | T | G |  | 15 | 13 |  | 11 |  | 9 |
| H15 | h33 | # | · | · | · | · | · |  | C | A | · | · | · | · | · | · | · | · | · | T | · | · |  | · | · | A | · | · | · | · | · |  | 10 | 18 |  | 10 |  | 9 |
|  | h34 | # | · | · | · | · | · |  | C | A | · | · | · | · | · | · | · | · | · | T | · | · |  | · | · | A | · | · | · | · | · |  | 10 | 17 |  | 10 |  | 9 |
|  | h35 | # | · | · | · | · | · |  | C | A | · | · | · | · | · | · | · | · | · | T | · | · |  | · | · | A | · | · | · | · | · |  | 10 | 16 |  | 10 |  | 9 |
| H16 | h36 | # | · | · | · | · | · |  | C | A | · | - | · | · | · | · | · | · | · | · | · | · |  | · | · | · | · | · | - | · | · |  | 10 | 15 |  | 11 |  | 9 |
| H17 | h37 | # | · | · | · | · | · |  | C | · | · | · | · | · | · | · | · | · | · | · | · | · |  | · | · | · | · | · | · | · | · |  | 10 | 14 |  | 11 |  | 9 |
|  | h38 | # | · | · | · | · | · |  | C | · | · | · | · | · | · | · | · | · | · | · | · | · |  | · | · | · | · | · | · | · | · |  | 10 | 12 |  | 11 |  | 9 |
|  | h39 | # | · | · | · | · | · |  | C | · | · | · | · | · | · | · | · | · | · | · | · | · |  | · | · | · | · | · | · | · | · |  | 10 | 12 |  | 10 |  | 9 |
| H18 | h40 | # | - | · | G | T | A |  | C | A | · | · | · | C | A | T | G | · | T | · | G | A |  | A | · | A | C | A | · | T | G |  | 16 | 13 |  | 11 |  | 10 |
|  | h41 | # | - | · | G | T | A |  | C | A | · | · | · | C | A | T | G | · | T | · | G | A |  | A | · | A | C | A | · | T | G |  | 15 | 12 |  | 12 |  | 10 |
| H19 | h42 | # | - | · | G | T | A |  | C | A | · | · | · | C | A | T | G | · | T | · | G | A |  | A | · | A | C | A | · | T | G |  | 15 | 12 |  | 11 |  | 10 |
| H20 | h43 | # | - | · | G | T | A |  | C | A | · | · | · | C | A | T | G | C | T | · | G | A |  | A | · | A | C | A | · | T | G |  | 16 | 14 |  | 11 |  | 10 |

All sequences are compared to the reference haplotype H1.

Note: * indicates sequneces ‘GAATCAGACAAA’; # indicates sequneces ‘TTTGTCTGATTC’; φ indicates sequneces ‘ATTCCATA’; § indicates sequneces ‘ACTCACAGG’; Dashes indicate missing nucleotides. H1-H20 refers to 20 haplotypes excluding the four SSR loci; h1 to h44 referred to haplotypes including all the SSR variation identified in three cpDNA sequences. A-453: (A)n; A-557: (T)n; B-634: (T)n; C-389: (A)n.
