# Supplementary material for: New insight into the phylogeographic pattern of Liriodendron chinense (Magnoliaceae) revealed by chloroplast DNA: east–west lineage split and genetic mixture within western subtropical China
Source: PeerJ. 2019 Feb 1;7:e6355. doi: 10.7717/peerj.6355 (PMC6361005; doi:10.7717/peerj.6355)
Supplement: Supplemental Information 2 — The size of each bubble was consistent with relative value of haplotype diversity, and the hollow circles represent the value of 0. [file peerj-07-6355-s002.docx]

Figure S1. Bubble diagram of population haplotype diversity (*h*) of *Liriodendron chinense* in western mountain region. The size of each bubble was consistent with relative value of haplotype diversity, and the hollow circles represent the value of 0.
