# Supplementary material for: New insight into the phylogeographic pattern of Liriodendron chinense (Magnoliaceae) revealed by chloroplast DNA: east–west lineage split and genetic mixture within western subtropical China
Source: PeerJ. 2019 Feb 1;7:e6355. doi: 10.7717/peerj.6355 (PMC6361005; doi:10.7717/peerj.6355)
Supplement: Supplemental Information 3 — K refers to the number of predefined groups used in the analyses. [file peerj-07-6355-s003.docx]

Figure S2. Results of spatial analysis of molecular variance analysis (SAMOVA, *K*=2-20) on *Liriodendron chinense* populations in subtropical China. *K* refers to the number of predefined groups used in the analyses.
