# Supplementary material for: New insight into the phylogeographic pattern of Liriodendron chinense (Magnoliaceae) revealed by chloroplast DNA: east–west lineage split and genetic mixture within western subtropical China
Source: PeerJ. 2019 Feb 1;7:e6355. doi: 10.7717/peerj.6355 (PMC6361005; doi:10.7717/peerj.6355)
Supplement: Supplemental Information 4 — The x-axis measures time in millions of years and the y-axis is the scaled effective population size. The thick solid line is the mean estimate, and the gray areas show the 95% HPD limits. (a) Whole range; (b) East lineage; (c) Southwest subclade; (d) Northwest subclade. [file peerj-07-6355-s004.docx]

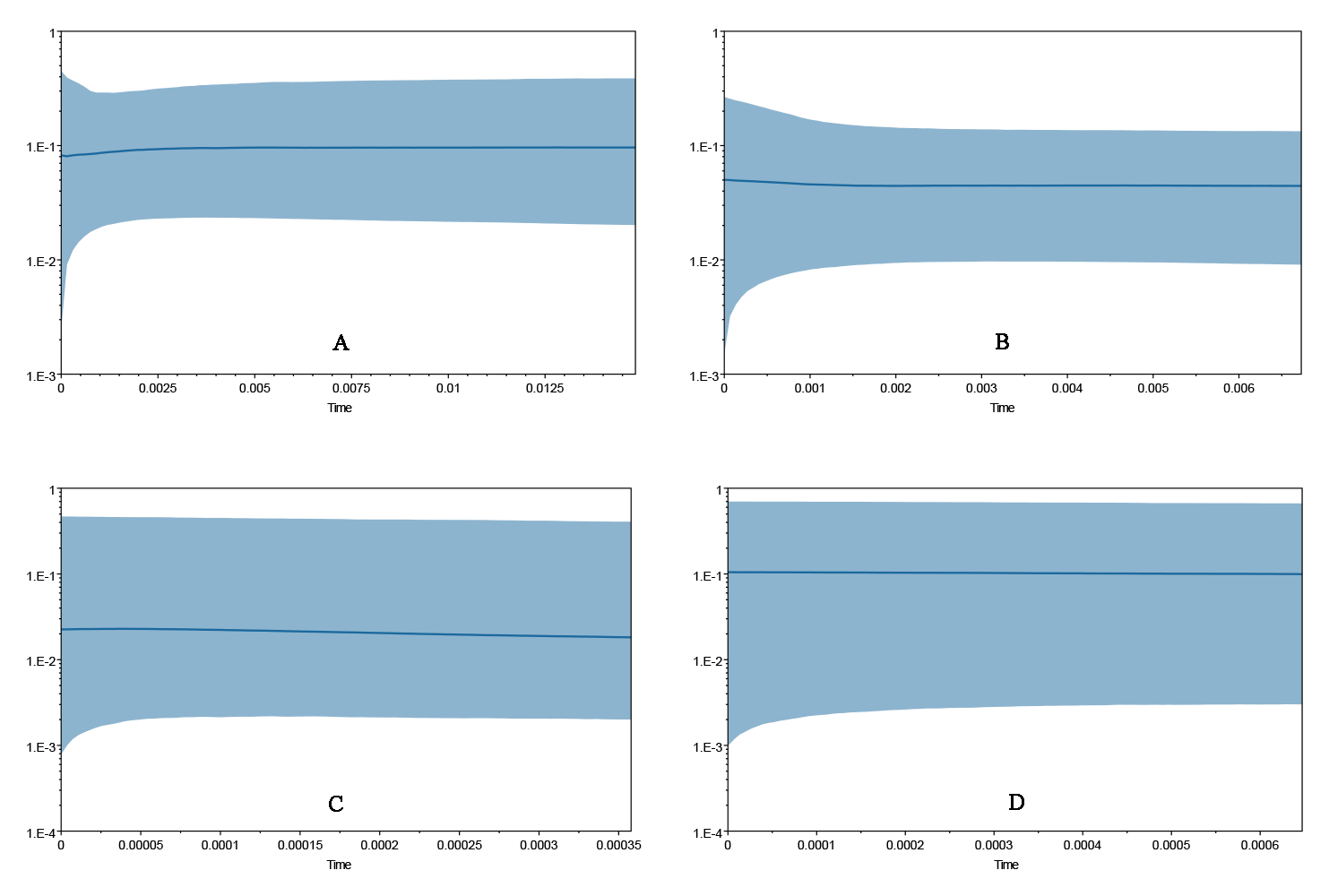


Figure S3. Bayesian skyline plot showing the estimated effective population size by region. The x-axis measures time in millions of years and the y-axis is the scaled effective population size. The thick solid line is the mean estimate, and the gray areas show the 95% HPD limits. (A) Whole range; (B) East lineage; (C) Southwest subclade; (D) Northwest subclade.
